# *Chop* deficiency prevents UUO-induced renal fibrosis by attenuating fibrotic signals originated from Hmgb1/TLR4/NF*κ*B/IL-1*β* signaling

**DOI:** 10.1038/cddis.2015.206

**Published:** 2015-08-06

**Authors:** M Zhang, Y Guo, H Fu, S Hu, J Pan, Y Wang, J Cheng, J Song, Q Yu, S Zhang, J-F Xu, G Pei, X Xiang, P Yang, C-Y Wang

**Affiliations:** 1 The Center for Biomedical Research, Tongji Hospital, Tongji Medical College, Huazhong University of Science and Technology, Wuhan, China; 2 Department of Clinical Immunology, Institute of Laboratory Medicine, Guangdong Medical College, Dongguan, China; 3 Department of Nephrology, Tongji Hospital, Tongji Medical College, Huazhong University of Science and Technology, Wuhan, China; 4 Department of Emergency Medicine, Institute of Emergency Medicine and Rare Diseases, The Second Xiangya Hospital, Central South University, Changsha, China

## Abstract

Renal fibrosis, particularly tubulointerstitial fibrosis is considered to be the final manifestation of almost all chronic kidney diseases (CKDs). Herein we demonstrated evidence that CHOP-related ER stress is associated with the development of renal fibrosis in both CKD patients and unilateral ureteral obstruction (UUO)-induced animals, and specifically, mice deficient in *Chop* were protected from UUO-induced renal fibrosis. Mechanistic studies revealed that loss of *Chop* protected tubular cells from UUO-induced apoptosis and secondary necrosis along with attenuated Hmgb1 passive release and active secretion. As a result, *Chop* deficiency suppressed Hmgb1/TLR4/NF*κ*B signaling, which then repressed UUO-induced IL-1*β* production. Consequently, the IL-1*β* downstream Erk1/2 activity and its related c-Jun transcriptional activity were reduced, leading to attenuated production of TGF-*β*1 following UUO insult. It was further noted that reduced IL-1*β* production also inhibited UUO-induced PI3K/AKT signaling, and both of which ultimately protected mice from UUO-induced renal fibrosis. Together, our data support that suppression of CHOP expression could be a viable therapeutic strategy to prevent renal fibrosis in patients with CKDs.

Renal fibrosis, featured by glomerulosclerosis and tubulointerstitial fibrosis, is generally thought to be the final manifestation of a wide variety of chronic kidney diseases (CKDs).^1, 2^ Given that renal fibrosis is an unavoidable consequence caused by the excessive accumulation of extracellular matrix virtually in the setting of patients with different types of CKDs, it serves as a pathological marker relevant to end-stage renal failure, a condition that requires dialysis or renal transplantation for maintaining the life of patients.^3^ There is compelling evidence that mesangial and fibroblast activation, tubular epithelial-to-mesenchymal transition (EMT), inflammatory (monocyte, macrophage and T cell) infiltration and apoptosis are common cellular events leading to renal fibrosis.^4, 5, 6, 7^ Past extensive studies have consistently demonstrated the essential role of TGF-*β* and its downstream Smad signaling had in the pathogenesis of renal fibrosis,^8, 9^ but the causative factors that trigger TGF-*β* expression and the molecular mechanisms that initiate the above-described cellular events are yet to be fully addressed.

Endoplasmic reticulum (ER) is crucial for protein biosynthesis, folding, trafficking and modification, and therefore, disturbances of ER homeostasis by extracellular stimuli such as oxidative stress would affect protein folding and cause ER stress. Given that ER stress-associated apoptosis modulates organ remodeling after insult, ER stress has been demonstrated with implications in the pathogenesis of cardiac and hepatic fibrosis.^10, 11^ In contrast, the impact of ER stress in the pathoetiology of renal fibrosis, however, is not yet to be clearly elucidated. More recently, Chiang *et al.*^12^ provided feasible evidence suggesting the involvement of ER stress in renal apoptosis and fibrosis, and studies in albumin-overloaded renal tubular cells further revealed that suppression of oxidative stress attenuates EMT and reduces ER stress.^13^ Based on these observations, we first conducted studies in CKD patients to demonstrate the presence of ER stress during the course of renal fibrosis, unilateral ureteral obstruction (UUO) was then induced in mice deficient in CCAAT/enhancer-binding protein (C/EBP) homologous protein (*Chop*) to dissect the cellular and molecular events relevant to ER stress in renal fibrosis. It was noted that loss of *Chop* provided remarkable protection for mice against UUO-induced renal fibrosis. Altered Chop expression rendered tubular cells undergoing apoptosis and secondary necrosis along with Hmgb1 passive release, which then recruited immune cells such as macrophages into the damaged site along with active secretion of copious amount of Hmgb1. Extracellular Hmgb1 thus bound to TLR4, and by which, it activated MyD88-NF*κ*B pathway to enhance IL-1*β* expression, which in turn promoted TGF-*β*/Smad2/3 and Pi3k/Akt signaling to exacerbate renal fibrosis.

## Results

### Patients with renal fibrosis manifest altered CHOP expression along with macrophage infiltration

**Figure 1:**
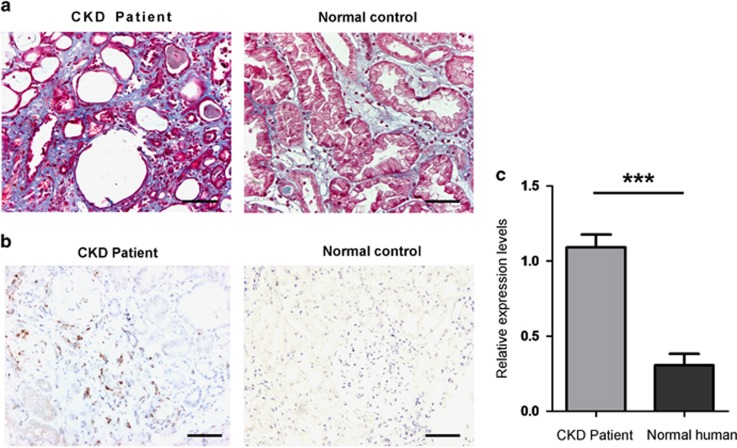
Results for analysis of renal biopsy samples from CKD patients. (**a**) Masson staining showing tubulointerstitial collagen deposition in CKD patients. (**b**) Immunohistochemical staining of CD68, a marker for macrophage in biopsy sections. (**c**) Real-time PCR analysis of CHOP expression in renal biopsy samples. The relative expression levels for CHOP were normalized by GAPDH, and a total of six patients were analyzed. Bar=100 *μ*m; ***P<0.001

We first sought to examine the relevance of CHOP expression in patients with renal fibrosis. Renal biopsy tissues originated from patients with CKD were subjected to Masson staining to screen patients with the onset of renal fibrosis. After screening a number of patients, six CKD patients were characterized with a significant collagen deposition (Figure 1a), indicating the onset of renal fibrosis. Biopsy sections from those six patients were next stained for CD68, a marker for macrophages. A marked macrophage infiltration was noted in all of those patients as compared with that of normal controls (Figure 1b). Next, we examined CHOP expression by real-time PCR. A threefold higher CHOP expression was detected in patients with renal fibrosis as compared with that of control subjects (Figure 1c).

### ER stress occurs during the course of UUO-induced renal fibrosis

**Figure 2:**
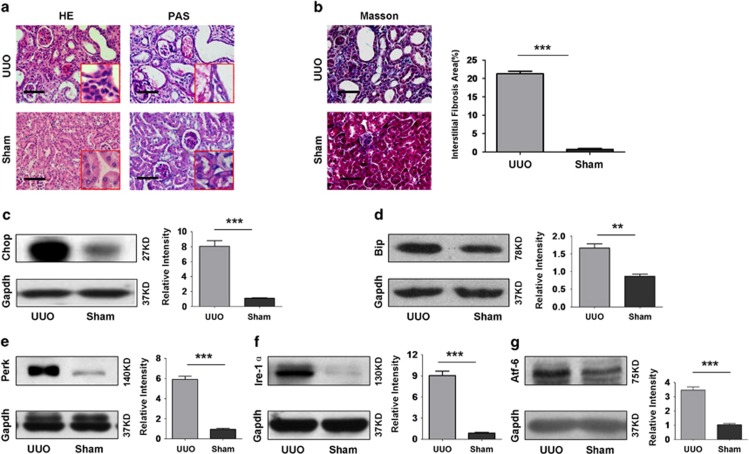
Analysis of *Chop* expression and ER stress markers in the kidneys with UUO-induced fibrosis. WT mice were first subjected to UUO procedures and then killed after day 14 of UUO induction. Sham-operated mice were served as controls. (**a**) H&E (left panel) and PAS (right panel) staining showing pathological changes following UUO induction. (**b**) Masson's trichrome staining showing collagen deposition in UUO-induced kidneys. (**c**–**g**) Western blot analysis of Chop (**c**) and ER stress markers Bip (**d**), Perk (**e**), Ire-1*α* (**f**) and Atf-6 (**g**). **P<0.01 and ***P<0.001

To confirm the above observation in patients, we conducted UUO in mice to induce renal fibrosis, and mice undergone similar surgical process but without UUO were served as controls (Sham). The mice were killed 14 days after UUO induction to examine the onset of renal fibrosis. It was noted that UUO-induced hydronephrosis, pyelectasis and renal tubular expansion as determined by H&E and periodic acid–Schiff (PAS) staining (Figure 2a). Indeed, UUO induced the onset of renal fibrosis as manifested by the significant collagen deposition (Figure 2b). We then checked the presence of ER stress, and a sevenfold increase for Chop expression was noted in UUO-induced mice as compared with that of control mice (Figure 2c and Supplementary Figure 1A). Notably, a significant accumulation for immunoglobulin-binding protein (Bip), an initiator for mediating unfolded protein response, was characterized in UUO-induced kidneys (Figure 2d and Supplementary Figure 1B). Similarly, the three downstream ER stress sensors, protein kinase RNA-like kinase (Perk; Figure 2e and Supplementary Figure 1C), inositol-requiring protein-1*α* (Ire-1*α*; Figure 2f and Supplementary Figure 1D), and activating transcription factor 6 (Atf-6; Figure 2g and Supplementary Figure 1E) were markedly increased in UUO-induced kidneys. Collectively, our data support that CHOP-related ER stress is a characteristic feature during the course of renal fibrosis.

### *Chop* deficiency attenuates UUO-induced renal fibrosis

**Figure 3:**
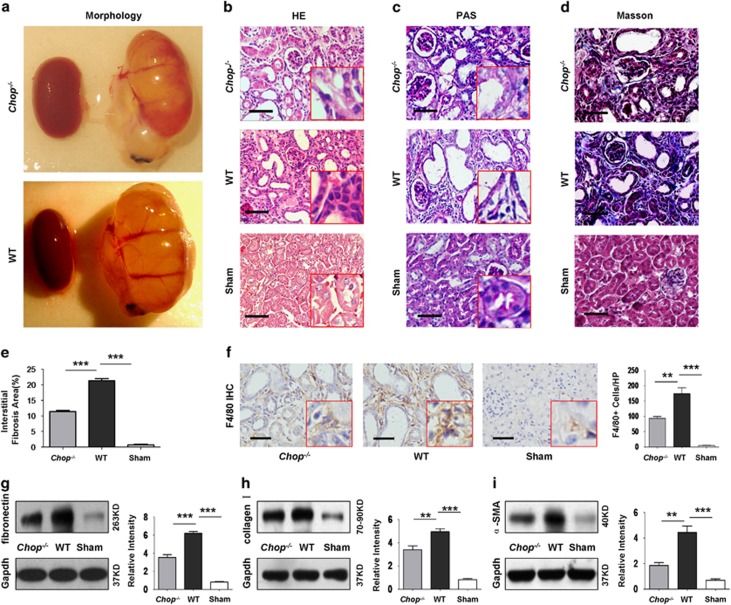
Loss of *Chop* provides protection for mice against UUO-induced renal fibrosis. Both WT and *Chop*^*−/−*^ mice were subjected to UUO induction for 14 days and then killed for comparative analysis of renal fibrosis and ER stress. Similarly, Sham-operated mice were served as controls. (**a**) *Chop* deficiency attenuated UUO-induced renal morphological changes. (**b**) H&E staining of renal sections for analysis of inflammatory infiltration. (**c**) PAS staining for assessment of the integrity tubular brush border and tubular dilation. (**d**) Masson staining for analysis of collagen deposition. (**e**) Semiquantitative analysis of tubulointerstitial fibrotic area. (**f**) F4/80 staining for analysis of macrophage infiltration. (**g**–**i**) Western blot analysis of fibrotic markers fibronectin (**g**), collagen I (**h**) and *α*-SMA (**i**). Bar=100 *μ*m. **P<0.01; ***P<0.001

To further address the impact of ER stress on renal fibrosis, we induced renal fibrosis by UUO in *Chop*-deficient and wild-type (WT) mice as above. It was interestingly noted that the size of UUO-induced kidneys in *Chop*^*−/−*^ mice was much smaller than that of WT mice (Figure 3a). In line with this observation, the severity for inflammatory infiltration (Figure 3b), loss of integrality for tubular brush border and renal tubular dilation (Figure 3c) was significantly attenuated in *Chop*^*−/−*^ mice as compared with that of WT mice. Particularly, Masson staining demonstrated much lower severity for interstitial fibrosis in *Chop*^*−/−*^ mice (Figures 3d and e) along with a significant reduction for the number of infiltrated macrophages (Figure 3f). Indeed, western blot analysis indicated significantly lower levels for the expression of fibrogenic markers fibronectin (Figure 3g), collagen (Figure 3h) and *α*-SMA (Figure 3i) in UUO-induced *Chop*^*−/−*^ kidneys, and consistent results were obtained by RT-PCR analysis of UUO-induced renal lysates (Supplementary Figures 2A, B and C). Taken together, our data support that loss of *Chop* provides protection for mice against UUO-induced renal fibrosis.

### *Chop* deficiency represses ER stress in UUO-induced renal fibrosis

**Figure 4:**
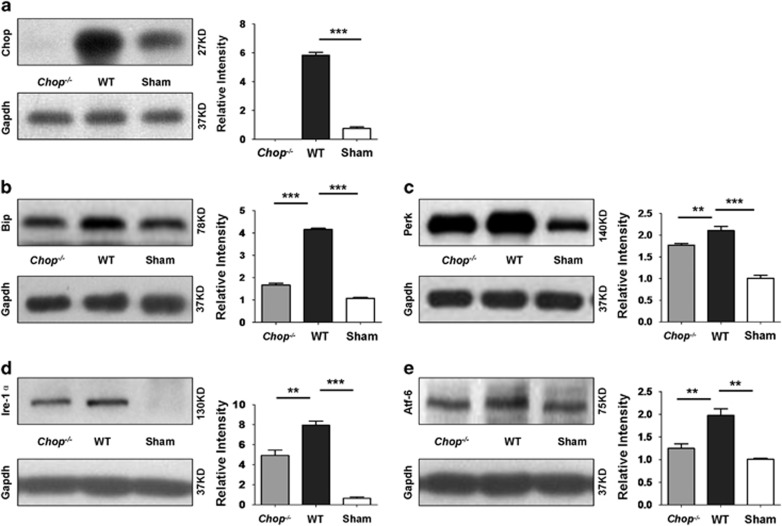
*Chop* deficiency attenuates UUO-induced ER stress in the kidneys. Renal lysates after 14 days of UUO induction were prepared and subjected to comparative western blot analysis of ER stress markers Chop (**a**), Bip (**b**), Perk (**c**), Ire-1*α* (**d**) and Atf-6 (**e**). Six mice were studied in each group, and relative expression levels for each target were normalized as a ratio to Gapdh. **P<0.01; ***P<0.001

Given the role of CHOP had in ER stress,^14^ we then compared the expression of ER stress markers between *Chop*^*−/−*^ and WT mice in the setting of UUO-induced renal fibrosis. As expected, Chop was absent in *Chop*^*−/−*^ mice, but similar as the above studies that UUO-induced renal fibrosis was associated with a marked increase for Chop expression (Figure 4a and Supplementary Figure 3A). Importantly, the expression of Bip was reduced by 1.5-fold in *Chop*^*−/−*^ mice as compared with that of WT mice after day 14 of UUO induction (Figure 4b and Supplementary Figure 3B). Similarly, a significant reduction for the expression of Perk (Figure 4c and Supplementary Figure 3C), Ire-1*α* (Figure 4d and Supplementary Figure 3D) and Atf-6 (Figure 4e and Supplementary Figure 3E) was also noted in *Chop*^*−/−*^ mice. These results suggest that loss of *Chop* attenuated ER stress in the setting of UUO-induced renal fibrosis.

### Loss of *Chop* protects tubular cells from UUO-induced apoptosis

**Figure 5:**
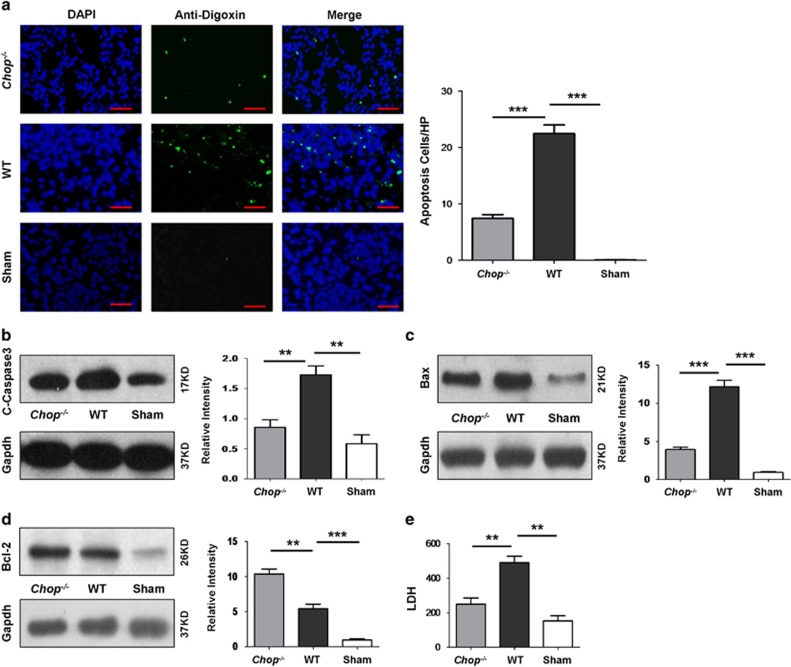
Loss of *Chop* protects UUO-induced tubular apoptosis and secondary necrosis. (**a**) TUNEL assay for analysis of tubular apoptosis. UUO-induced extensive tubular apoptosis in WT mice, which was significantly reduced in *Chop*^*−/−*^ mice. (**b**–**d**) Western blot analysis for pro-apoptotic proteins caspase 3 (**b**) and Bax (**c**), and anti-apoptotic protein Bcl-2 (**d**). Loss of Chop inhibited UUO-induced pro-apoptotic caspase 3 and Bax, but enhanced anti-apoptotic Bcl-2 expression. (**e**) Analysis of LDH activity for assessing secondary necrosis. Bar=100 *μ*m. **P<0.01; ***P<0.001

As tubular apoptosis is a crucial event for the initiation of renal fibrosis,^7^ we then conducted TUNEL assay to assess tubular apoptosis. As expected, tubular apoptosis is almost undetectable in Sham-operated mice. However, high levels of tubular apoptosis were characterized in WT mice upon UUO induction, and *Chop*^*−/−*^ mice manifested a twofold reduction for tubular apoptosis (Figure 5a). To confirm this observation, we further examined the expression of two apoptotic markers, caspase 3 and Bax. Indeed, *Chop* deficiency attenuated UUO-induced caspase 3 expression by onefold (Figure 5b) and Bax expression by twofold (Figure 5c and Supplementary Figure 4A). In sharp contrast, loss of *Chop* enhanced the expression of anti-apoptotic Bcl-2 by onefold (Figure 5d and Supplementary Figure 4B). Given that altered apoptosis is generally associated with secondary necrosis,^15^ we thus next checked lactate dehydrogenase (LDH) activity for the presence of necrosis. UUO induction resulted in a 2.3-fold increase for LDH activity as compared with that of Sham-operated mice (Figure 5e), indicating that secondary necrosis did occur following UUO insult. Importantly, *Chop*^*−/−*^ mice manifested an 80% reduction for LDH activity as compared with that of WT mice (Figure 5e), suggesting that *Chop* deficiency protected tubular cells undergoing UUO-induced apoptosis, thereby prevented the occurrence of secondary necrosis.

### Chop regulates UUO-induced Hmgb1 passive release and active secretion

**Figure 6:**
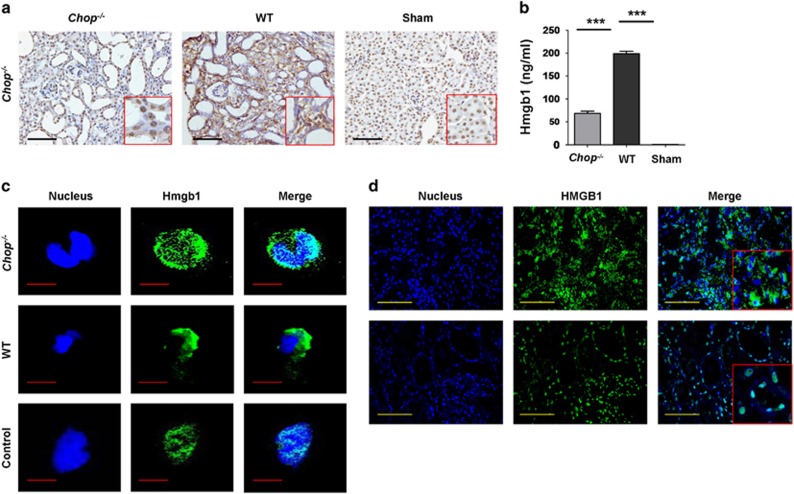
Analysis of HMGB1 subcellular location in fibrotic renal sections and LPS-stimulated macrophages. (**a**) Immunohistochemical staining showing Hmgb1 passive release in the kidneys following UUO induction. (**b**) Analysis of serum Hmgb1 levels after 14 days of UUO induction. (**c**) Loss of *Chop* attenuated LPS-induced Hmgb1 secretion in macrophages as manifested by the reduced Hmgb1 cytoplasmic translocation. ***P<0.001. (**d**) Immunostaining of HMGB1 in renal biopsy samples originated from CKD patients and control subjects. HMGB1 was stained in green and nucleus was counterstained in blue by DAPI. Inset pictures (x400) were included to show HMGB1 subcellular location. Bar=100 *μ*m

It has been well demonstrated that necrosis secondary to apoptosis would result in the passive release of Hmgb1, an innate alarmin, into the extracellular milieu.^15, 16^ Analysis of renal sections derived from Sham-operated mice revealed that Hmgb1 was solely localized in the nucleus. On the contrary, Hmgb1 was also detected in the cytoplasm of damaged cells and extracellular areas adjacent to the damaged cells (Figure 6a), indicating Hmgb1 passive release following UUO insult. However, the passively released Hmgb1 was significantly attenuated in mice deficient in *Chop* (Figure 6a).

We next checked the impact of *Chop* deficiency on Hmgb1 secretion. We first examined Hmgb1 serum levels, and *Chop*^*−/−*^ mice manifested a twofold lower serum Hmgb1 as compared with that of WT mice following UUO induction (Figure 6b), suggesting that Chop also regulates Hmgb1 active secretion. Peritoneal macrophages were next prepared from both WT and *Chop*^*−/−*^ mice and then subjected to lipopolysaccharide (LPS) stimulation for 24 h, followed by immunostaining of Hmgb1 subcellular location. Hmgb1 was only detected in the nucleus of unstimulated WT macrophages, whereas Hmgb1 was almost completely translocated into the cytoplasm following LPS stimulation, but LPS-induced Hmgb1 cytoplasmic translocation was markedly abrogated in *Chop*^*−/−*^ macrophages (Figure 6c), confirming that Chop also regulates Hmgb1 active secretion

The above observations prompted us to check biopsy sections from CKD patients again for analysis of HMGB1 subcellular location and expression levels. Similar as the data from UUO-induced mice, biopsy sections originated from CKD patients manifested significantly higher HMGB1 fluorescent intensity than that of renal sections derived from normal subjects (Figure 6d). More importantly, HMGB1 was only detected in the nucleus of sections from normal subjects, whereas HMGB1 was also detected in the cytoplasm of sections from CKD patients (Figure 6d, inset picture). Together, those data indicate that renal fibrosis in CDK patients are also characterized by the enhanced HMGB1 expression along with its cytoplasmic translocation.

### Loss of *Chop* abrogates Hmgb1/TLR4/NF*κ*B signaling

**Figure 7:**
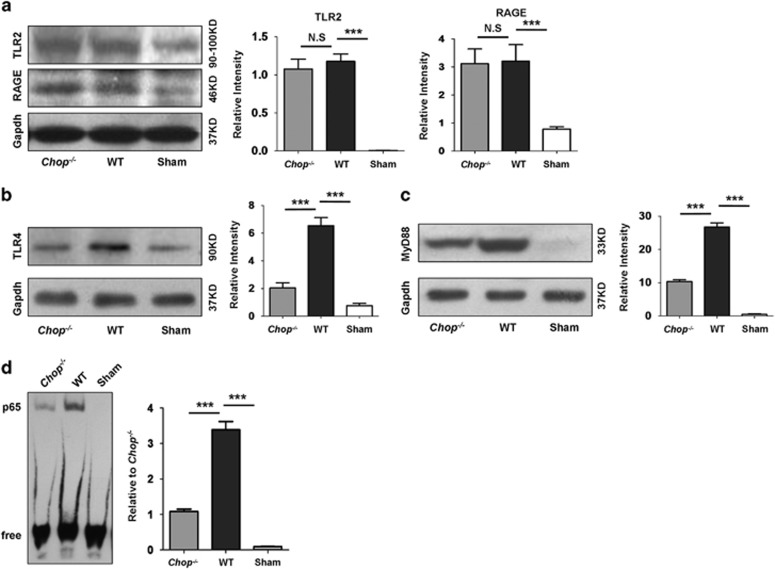
*Chop* deficiency attenuates UUO-induced TLR4-NF*κ*B signaling. (**a**) *Chop* deficiency does not affect UUO-induced TLR2 and Rage expression in the kidneys. UUO induced a significant upregulation for both TLR2 and RAGE, but no expression difference was noted between WT and *Chop*^*−/−*^ mice. (**b**) Loss of *Chop* significantly reduced UUO-induced TLR4 expression in the kidneys. (**c**) MyD88, an HMGB1/TLR4 downstream signaling molecule, was also repressed in *Chop*^*−/−*^ mice following UUO induction. (**d**) *Chop* deficiency repressed UUO-induced NF*κ*B DNA-binding activity. UUO induced significantly higher levels of NF*κ*B DNA-binding activity as compared with that of Sham-operated mice, whereas a significantly reduced DNA-binding activity was noted *Chop*^*−/−*^ mice. Bar=100 *μ*m. ***P<0.001

Upon its release into extracellular milieu, Hmgb1 would act as a potent inflammatory mediator through receptors TLR2, TLR4 and RAGE.^15, 16^ Interestingly, although UUO insult induced a significant upregulation for TLR2 and RAGE in the kidneys from both WT and *Chop*^*−/−*^ mice, but no perceptible difference in terms of their expression levels were noted between WT and *Chop*^*−/−*^ mice (Figure 7a). In contrast, a twofold higher TLR4 expression was noted in UUO-induced WT mice as compared with that of *Chop*^*−/−*^ mice (Figure 7b), indicating that Hmgb1 probably exerts its inflammatory effect predominantly through TLR4. To further confirm this conclusion, we examined the expression of MyD88, a crucial signaling molecule downstream of TLR4. Indeed, WT mice manifested a 1.5-fold higher MyD88 expression in the UUO-induced kidneys as compared with that of *Chop*^*−/−*^ mice (Figure 7c). We then assessed DNA-binding activity for NF*κ*B, a downstream transcription factor relevant to inflammatory response and fibrosis. Consistently, loss of *Chop* attenuated UUO-induced NF*κ*B-binding activity by twofold (Figure 7d).

### *Chop* deficiency represses the axis of TGF-*β*/Smad2/3 and Pi3k/Akt signaling

**Figure 8:**
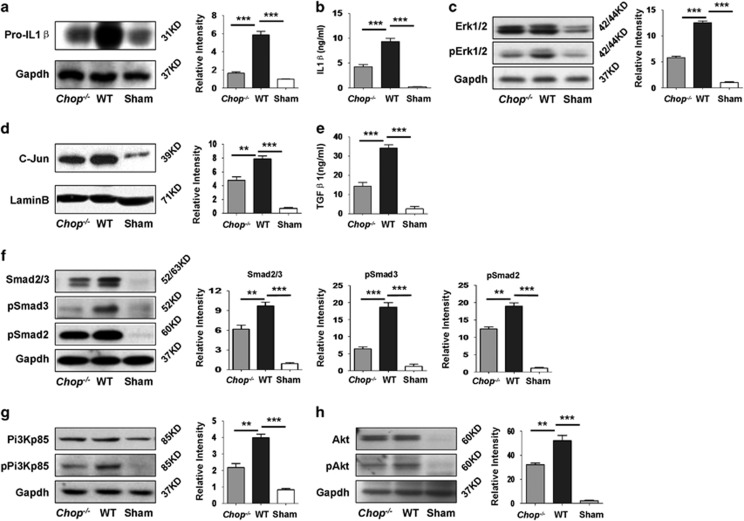
*Chop* deficiency attenuates fibrotic signals originated from IL-1*β* downstream TGF-*β*1/Smad2/3 and PI3k/AKT signaling. (**a**) *Chop* deficiency inhibited UUO-induced pro-IL-1*β* expression in the kidneys. (**b**) ELISA analysis of serum IL-1*β* in mice following 14 days of UUO induction. Similar as above, the production of IL-1*β* in the serum was significantly reduced in *Chop*^*−/−*^ mice. (**c** and **d**) Western blot analysis of IL-1*β* downstream Erk1/2 activity (**c**) and c-Jun expression (**d**). The reduced IL-1*β* because of *Chop* deficiency attenuated Erk1/2 activation as manifested by the lower levels of pErk1/2, and as a result, the expression of its downstream TGF-*β*1 transcription factor, c-Jun, was significantly reduced. (**e**) ELISA analysis of TGF-*β*1 in UUO-induced renal lysates. In line with reduced c-Jun expression in *Chop*^*−/−*^ kidneys, the production of renal TGF-*β*1 in *Chop*^*−/−*^ mice was significantly lower as compared with that of WT mice. (**f**) Western blot analysis of TGF-*β*1 downstream Smda2/3 signaling. In consistent with the above data, both total Smad2/3 and p-Smad2 and three were significantly reduced in *Chop*^*−/−*^ kidneys following UUO induction. (**g** and **h**) The impact of *Chop* deficiency on PI3k/AKT signaling. Other than regulating TGF-*β* signaling, IL-1*β* also promotes Pi3k activity to exacerbate the development of renal fibrosis. Consistently, the phosphorylated levels for Pi3kp85 subunit (pPi3kp85) (**g**) and Akt (pAkt) (**h**) were significantly reduced. **P<0.01; ***P<0.001

To address the mechanisms by which *Chop* deficiency attenuates UUO-induced renal fibrosis, we next assessed IL-1*β* expression, a cytokine transcribed by NF*κ*B, which is essential for the initiation of renal fibrosis.^7^ In consistent with the attenuated NF*κ*B transcriptional activity, *Chop*^*−/−*^ mice were featured by a 2.6-fold reduction of pro-IL1*β* expression in the kidneys following UUO induction (Figure 8a). Similarly, ELISA analysis revealed a 1.3-fold decrease for the production of serum IL-1*β* in UUO-induced *Chop*^*−/−*^ mice as compared with that of WT mice (Figure 8b).

Next, we check the impact for the attenuated IL-1*β* production on pro-fibrotic factor TGF-*β*1 transcription. It was noted that *Chop* deficiency did not affect the expression of total Erk1/2 following UUO induction, whereas the activated Erk1/2 (pErk1/2) was reduced by onefold as compared with that of WT mice (Figure 8c), which resulted in a significant decrease for the expression of transcription factor c-Jun (Figure 8d) along with a 1.5-fold decrease for the production of TGF-*β*1 in UUO insulted kidneys (Figure 8e). As a result, the activity for TGF-*β* downstream Smad2/3 was significantly attenuated (Figure 8f). It is noteworthy that IL-1*β* has also been found to promote renal fibrosis through the activation of PI3K/AKT pathway.^17^ Indeed, a significant reduction for the phosphorylated Pi3kp85 (pPi3kp85) (Figure 8g) and its downstream pAkt (Figure 8h) was noted. Taken all data together, our results suggest that loss of *Chop* attenuates the production of IL-1*β*, thereby preventing UUO-induced renal fibrosis not only directly through suppression of TGF-*β*/Smad2/3 signaling, but also by inhibition of Pi3k/Akt signaling, which has been demonstrated relevant to the initiation of EMT.^17^

## Discussion

Despite past extensive studies, the exact molecular mechanisms underlying renal fibrosis are yet to be fully addressed. Recent studies demonstrated feasible evidence suggesting a role for ER stress in the development of renal fibrosis in the setting of patients with CKDs.^18^ Given the role of CHOP had in the induction of ER stress, we thus hypothesized that CHOP may have a critical role in the pathogenesis of renal fibrosis secondary to CKDs. We first examined CHOP expression in biopsy samples of patients with renal fibrosis resulted from CKDs. Indeed, renal fibrosis was associated with a threefold increase for CHOP expression as compared with that of control subjects. To confirm this observation, we next conducted UUO in mice to induce renal fibrosis. Similar as the studies in CKD patients, renal fibrosis was associated with a sevenfold of Chop upregulation and, importantly, a significant increase for other ER stress markers such as Bip, Perk, Ire-1*α* and Atf-6 was also noted in UUO-induced mice. Together, these studies demonstrated strong evidence supporting that CHOP-related ER stress is implicated in the pathogenesis of renal fibrosis.

To dissect the exact impact of CHOP on the development of renal fibrosis, we conducted UUO in both WT and *Chop*^*−/−*^ mice and then examined the severity of renal fibrosis after 14 days of surgery. Remarkably, *Chop* deficiency significantly attenuated UUO-induced renal fibrosis as evidenced by the smaller size of the kidneys along with attenuated inflammatory infiltration, collagen deposition, reduced interstitial fibrotic area and repressed expression of fibrotic markers fibronectin, collagen and *α*-SMA. It was interestingly noted that loss of *Chop* significantly inhibited UUO-induced ER stress as manifested by the reduced expression of Bip, Perk, Ire-1*α* and Atf-6, which further supports the implication of CHOP-related ER stress in the development of renal fibrosis.

To address the molecular mechanisms by which *Chop* deficiency attenuates UUO-induced renal fibrosis, we first examined the impact of *Chop* deficiency on UUO-induced tubular apoptosis as UUO-induced ER stress in the tubular compartment is known to cause cell injury and death, which serves as the initial step to the development of renal fibrosis.^7^ Indeed, TUNEL analysis of renal sections revealed extensive tubular apoptosis following UUO induction, but loss of *Chop* attenuated UUO-induced tubular apoptosis by twofold. In line with this observation, the expression of major apoptotic effectors, caspase 3 and Bax, was significantly reduced in UUO-induced *Chop*^*−/−*^ mice as compared with that of WT mice. However, the expression of anti-apoptotic Bcl-2 was significantly increased in *Chop*^*−/−*^ mice. This result is consistent with previous studies that CHOP influences Bcl-2 expression translationally and transcriptionally.^19, 20^ Generally, extensive apoptosis is associated with secondary necrosis, we thus further examined LDH levels in renal lysates after day 14 of UUO induction. UUO-induced WT mice manifested 2.3-fold higher LDH activity as compared with that of Sham-operated mice, indicating that necrosis occurred secondary to extensive tubular apoptosis. However, significantly lower levels of LDH were noted in *Chop*^*−/−*^ mice than that of WT mice, demonstrating that loss of *Chop* suppressed UUO-induced ER stress, thereby prevented tubular cells undergoing apoptosis and secondary necrosis.

It has been well demonstrated that damaged or necrotic cells are associated with passive release of endogenous damage-associated molecular patterns, among which HMGB1 is one of the most extensively studied alarmins. HMGB1 was originally characterized as a DNA-binding protein implicated in the regulation of nucleosomal structure and stability, and binding of transcription factors to their cognate DNA sequences. More recently, HMGB1 was further recognized to be a potent innate alarmin that alerts the innate immune system to initiate host defense against invaded pathogens or reparative response to tissue or organ injury. Extracellular HMGB1 exerts not only paracrine activity, but also autocrine activity on the cells from which it is secreted to enhance chemotactic and adaptive immune responses.^15^ Indeed, immunohistochemical staining of renal sections originated from UUO-induced mice revealed Hmgb1 passive release into the extracellular milieu, whereas loss of *Chop* significantly attenuated Hmgb1 passive release. More importantly, *Chop* deficiency also significantly decreased Hmgb1 secretion from macrophages, and therefore, Hmgb1 serum levels were much lower in *Chop*^*−/−*^ mice than that of WT mice after UUO insult.

TLR2, TLR4 and RAGE are thus far the receptors characterized for extracellular Hmgb1. Interestingly, it seems that TLR4 was probably the primary receptor for Hmgb1 in our model of UUO-induced renal fibrosis as *Chop* deficiency did not affect UUO-induced expression of TLR2 and RAGE but attenuated TLR4 by twofold. In line with this notion, MyD88, its downstream signaling molecule, was also significantly reduced following UUO induction. Upon binding to its cognate receptor, Hmgb1 would activate NF*κ*B to transcribe inflammatory cytokines relevant to the initiation and progression of renal fibrosis. It is noteworthy that loss of *Chop* repressed UUO-induced NF*κ*B DNA-binding activity by twofold.

Importantly, other than infiltrated inflammatory cells such as macrophages, injured tubular cells also secrete proinflammatory cytokines such as IL-1 upon NF*κ*B activation.^21, 22^ As a result, we have noted a significant increase for pro-IL-1*β* expression in the kidneys along with the production of copious amount of IL-1*β* in the serum after day 14 of UUO induction. IL-1*β* has been recognized as a potent stimulator to promote Erk1/2 activation, and by which it induces the expression of c-Jun, a critical transcription factor for TGF-*β*1 expression.^23, 24^ Indeed, we detected significantly higher levels of pErk1/2 and enhanced c-Jun expression in UUO-induced kidneys as compared with that of Sham-operated kidneys. As a result, UUO-induced kidneys manifested 14-fold higher TGF-*β*1 production than that of Sham-operated kidneys. Given that *Chop* deficiency attenuated UUO-induced NF*κ*B activation, the production of IL-1*β* in UUO-induced *Chop*^*−/−*^ kidneys were significantly attenuated, and therefore, the downstream Erk1/2 signaling and c-Jun expression were markedly suppressed. Particularly, the production of TGF-*β*1 in UUO-induced *Chop*^*−/−*^ kidneys was reduced by 1.5-fold as compared with that of WT mice. Through the interaction with its receptors on epithelial cells, mesangial cells and fibroblasts, TGF-*β*1 can enhance the deposition of extracellular matrix proteins (e.g., collagens I, III and IV and fibronectin) to initiate and promote the development of renal fibrosis.^25, 26^ Of note, other than stimulating TGF-*β*1 secretion, there is also evidence indicating that IL-1*β* can activate PI3K/Akt signaling,^27^ and thereby enhancing renal fibrosis.^28^ Indeed, western blot analysis of renal lysates revealed a significant reduction for the activated pPi3kp85 and pAkt following UUO induction in *Chop*^*−/−*^ mice as compared with that of WT mice. Collectively, our data suggest that loss of *Chop* protects mice against UUO-induced renal fibrosis by preventing tubular apoptosis and secondary necrosis, and by which *Chop* deficiency represses Hmgb1 passive release and active secretion, which then inhibits Hmgb1/TLR4/NF*κ*B signaling along with attenuated production of IL-1*β*; whereas reduced IL-1*β* production following UUO insult would not only limit the secretion of TGF-*β* but also the activation of PI3K/AKT signaling, which then attenuates the initiation and progression of renal fibrosis.

In summary, the development of renal fibrosis is a complicated process, which involves a number of factors. In the current report, we only tackled the impact of CHOP-related ER stress on the pathology of UUO-induced renal fibrosis. Particularly, we investigated with focus for the impact of *Chop* deficiency on tubular apoptosis and secondary necrosis relevant to Hmgb1 passive release and active secretion. We demonstrated evidence that loss of *Chop* represses Hmgb1/TLR4 signaling following UUO induction, leading to repressed NF*κ*B transcriptional activity along with suppressed IL-1*β* production, which then reduces TGF-*β*1 production and Pi3K/Akt activity to attenuate the development of renal fibrosis. Although *Chop* deficiency did not affect UUO-induced TLR2 and RAGE expression, UUO induced a significant upregulation for both TLR2 and RAGE, indicating that additional factors other than CHOP-associated HMGB1 are also implicated in the pathogenesis of renal fibrosis. Future studies with focus on this aspect would be necessary to fully address the mechanisms underlying renal fibrosis in the setting of patients with CKDs.

## Materials and Methods

### Human subjects and animals

Renal biopsy samples from patients with CKD were provided by the Department of Nephrology of Tongji Hospital, whereas fresh normal renal tissues were provided by five patients who died from lung cancer. Informed consent was obtained from each subject and the study was approved by the Human Assurance Committee (HAC) in Tongji Hospital. CHOP-deficient mice and C57BL/6 mice (7 weeks old, 23–28 g) were purchased from the Jackson's Laboratory (Bar Harbor, ME, USA). All mice were housed in the experimental animal center at the Tongji Medical College, Huazhong University of Science and Technology with a 12/12-h light/dark cycle. After a minimum 1 week of acclimation, the mice (*n*=15 for each group) were subjected to surgical procedures for UUO. Briefly, the mice were anesthetized by pentobarbital, and the left ureter was isolated from the surrounding tissues, double ligated. The partial ureter between the two ligations was then cut by scissors, followed by injection of 500 *μ*l sterile normal saline into the abdominal cavity and the incision was next closed under aseptic condition. Mice undergone similar surgical procedures but without UUO were served as controls (Sham). All mice were killed at day 14 after UUO and kidneys were collected for experimental purpose. All studies were approved by the Animal Care and Use Committee (ACUC) in Tongji Hospital and conducted in accordance with NIH guidelines.

### Reagents

Antibodies for western blot analysis against Bip, Perk, Ire-1*α*, Chop, Pro-IL1*β*, p-Smad2, p-Smad3, p-P85 and p-P65 were purchased from Cell Signaling Technology (Danvers, MA, USA); Atf-6, fibronectin and *α*-SMA were purchased from Abcam (Cambridge, MA, USA); Myd88, c-Jun, TLR2, TLR4 and Gapdh were ordered from Santa Cruz Biotechnology (Santa Cruz, CA, USA); Collagen I was purchased from Chemicon International (Temecula, CA, USA); caspase 3 was derived from Abgent (San Diego, CA, USA); and Hmgb1 was purchased from Abnova (San Diego, CA, USA). For immunohistochemical analysis, antibody for CHOP was purchased from Santa Cruz Biotechnology; HMGB1 was derived from Epitomics, Inc. (Burlingame, CA, USA); and F4/80 was provided by AbD Serotec (Raleigh, NC, USA).

### Culture of peritoneal macrophages

Peritoneal macrophages were isolated from WT or *Chop*^*−/−*^ mice by intraperitoneal injection of 2 ml 4% sodium thioglycolate as reported.^29^ The isolated cells were cultured in 24-well plates with RPMI 1640 containing 10% fetal bovine serum (FBS) (Hyclone, Rockford, IL, USA). After 1 day of culture, the cells were stimulated with LPS for 24 h, followed by immunostaining of HMGB1 as previously described.^30^ The levels of translocation of HMGB1 in different groups were assessed by confocol microscopy.

### Histological and immunohistochemical analysis and immunostaining

Paraffin-embedded renal sections (3 *μ*m) were subjected to PAS and Masson trichome staining as previously reported.^31^ Tubulointerstitial fibrosis was analyzed by the percentage of fibrotic area in tubulointerstitial area using the Image pro plus software (Media Cybernetics, Rockville, MD, USA) in >10 randomly fields.^31^ For immunohistochemical analysis, the kidneys were fixed in 4% formaldehyde at 4 °C overnight and then embedded in paraffin. Tissue sections (3 *μ*m) were deparaffinized in xylene and rehydrated in graded alcohol. Endogenous peroxidase was blocked with 3% H_2_O_2_ and nonspecific proteins were blocked with 10% goat serum or rabbit serum for 30 min. The sections were then incubated with indicated primary antibodies at 4 °C for overnight, respectively, followed by incubation with an HRP-conjugated secondary antibody at room temperature for 30 min. The immunoreactive signals were developed with peroxidase substrate containing diaminobenzidine as reported.^32^ The sections were further counterstained with hematoxylin and then assessed under a microscope in a blinded manner by two pathologists. Immunostaining of HMGB1 in LPS-stimulated macrophages and biopsy sections from CDK patients were conducted as previously reported.^16^

### Western blot analysis

Renal tissues were homogenized in RIPA lysis buffer containing 1 mM phenylmethylsulfonyl fluoride (Amresco, Solon, OH, USA) and protease inhibitor cocktail (Roche, Indianapolis, IN, USA). Fifty micrograms of proteins was then separated by 12% SDS-PAGE and next electrophoretically transferred onto PVDF membranes. After blocking with 5% non-fat milk 1 h at room temperature, the membranes were probed with indicated primary antibodies at 4 °C overnight, followed by incubation with an HRP-conjugated secondary antibody. The reactive bands were visualized with ECL plus reagents as previously described.^33^ The relative expression levels for a particular target were assessed by densitometric analysis and normalized by GADPH using the Image J software (http://rsb.info.nih.gov/ij/) as instructed.

### Real-time quantitative RT-PCR

Total RNA extraction and cDNA were conducted using the ReverAid First Strand cDNA Synthesis Kit (Thermo Fisher Scientific, Waltham, MA, USA). Real-time PCR was performed with the LightCycler 480 system (Roche, Pleasanton, CA, USA) with primers specific for human CHOP, mouse Chop, Bip, Perk, Ire-1*α*, ATF6, fibronectin, collagen I, *α*-SMA, Bax and Bcl-2, respectively. The sequences for all primers are shown in Supplementary Table 1. Relative expression levels were normalized by GAPDH and calculated using the 2^−ΔΔCt^ approach as reported.^34^

### ELISA for cytokine production

The production of mouse IL-1*β* and TGF-*β* was determined using an ELISA Kit from BD Biosciences, (San Diego, CA, USA) and eBiosciences (San Diego, CA USA) using the established techniques,^35^ respectively.

### TUNEL assay

TUNEL assay was carried out using an ApopTag Plus Fluorescein In situ Apoptosis Detection Kit (Merck Millipore, Billerica, MA, USA) as previously reported.^15^ In brief, frozen sections (4 *μ*m) were fixed with 4% paraformaldehyde for 10 min at room temperature, followed by proteinase K treatment for 5 min. The sections were next incubated with TdT diluted in reaction buffer for 1 h at 37 °C, and then terminated by the addition of warmed stop buffer. After washes, the sections were incubated with an anti-digoxigenin conjugate for 30 min at 37 °C in the dark. Nucleus was counterstained with DAPI. Apoptosis was assessed by averaging the total number of apoptotic cells in 10 randomly selected fields at × 400 magnification of each section in a blinded manner. Three sections were examined for each mouse and three mice were included in each study group.

### Electrophoretic mobility shift assay (EMSA)

Nuclear proteins isolated from renal tissues were used for EMSA with a LightShift Chemiluminescent EMSA Kit (Thermo Scientific, Lafayette, CO, USA) using the established techniques.^36^ Biotin-labeled double-stranded oligonucleotides containing the NF*κ*B p65-binding site were used as a probe (forward: Biotin-5′-AGTTGAGGGGACTTTCCCAGGC-3′, reverse: Biotin-5′-GCCTGGGAAAGTCCCCTCAACT-3′ Biyuntian, Wuhan, China). A mixture of unlabeled probes was used as a negative control.

### Statistical analyses

All data are present as mean±S.E.M., and all *in vitro* experiments were conducted with at least three independent replications. A *P-*value of<0.05 was considered statistically significant. The SPSS 15.0 software (SPSS, Inc., Chicago, IL, USA) was used for statistical analysis using Student's *t*-test or one-way or two-way ANOVA where appropriate.^37^

## Abbreviations

CKD: chronic kidney diseases
UUO: unilateral ureteral obstruction
CHOP: CCAAT/enhancer-binding protein (C/EBP) homologous protein
ER: endoplasmic reticulum
HMGB1: high mobility group box 1
TLR4: Toll-like receptor 4
PI3K: phosphatidylinositol 3-kinase
AKT: protein kinase B
ERK: extracellular regulated protein kinases
TGF-*β*1: transforming growth factor-*β*1
NFκB: nuclear factor-κB
IL-1*β*: interleukin-1*β*
