# Supplementary figures and images for: Chop deficiency prevents UUO-induced renal fibrosis by attenuating fibrotic signals originated from Hmgb1/TLR4/NFκB/IL-1β signaling
Source: Cell Death Dis. 2015 Aug 6;6(8):e1847–. doi: 10.1038/cddis.2015.206 (PMC4558499; doi:10.1038/cddis.2015.206)

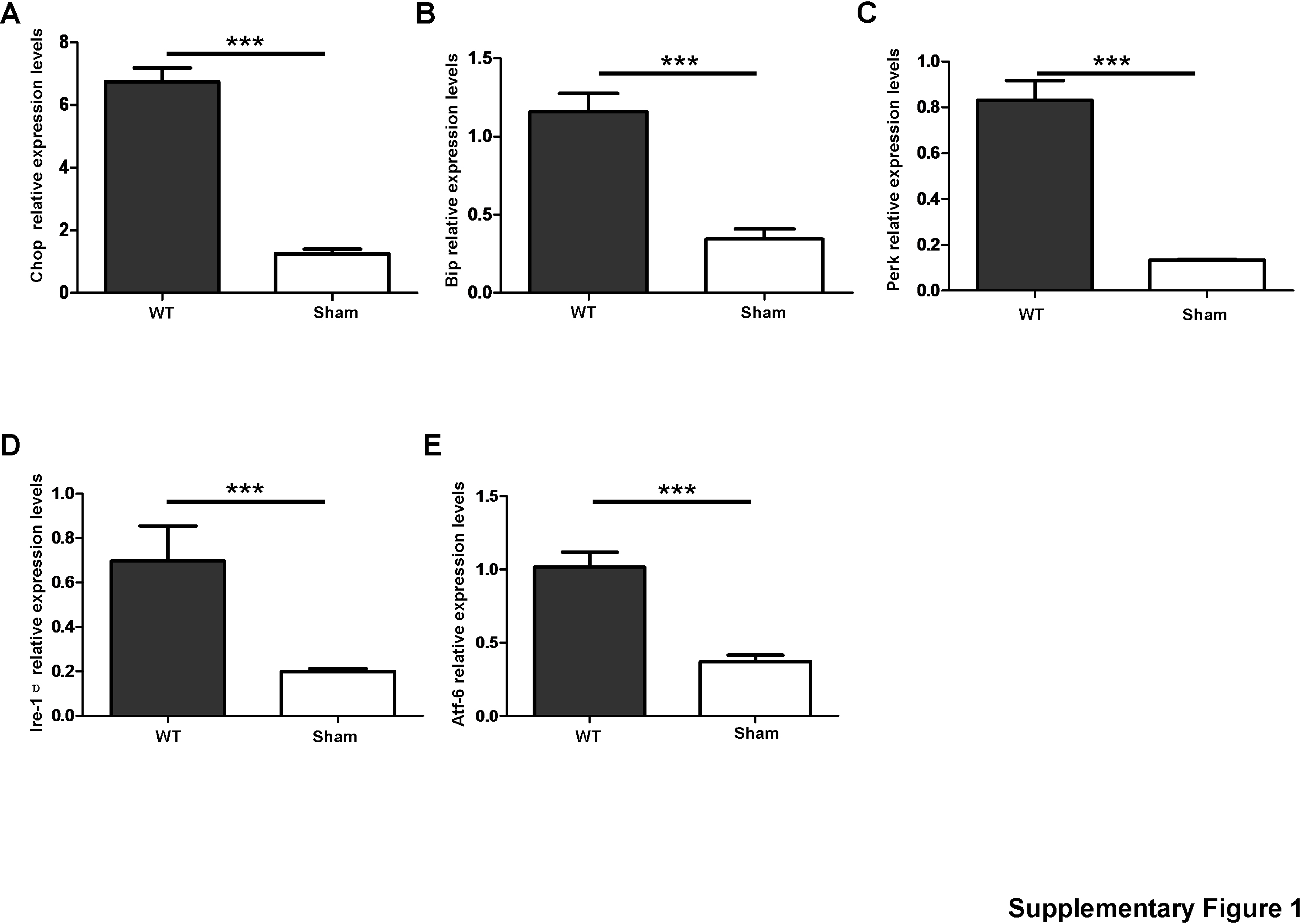

Supplement: Supplementary Figure 1 [file cddis2015206x1.tif]

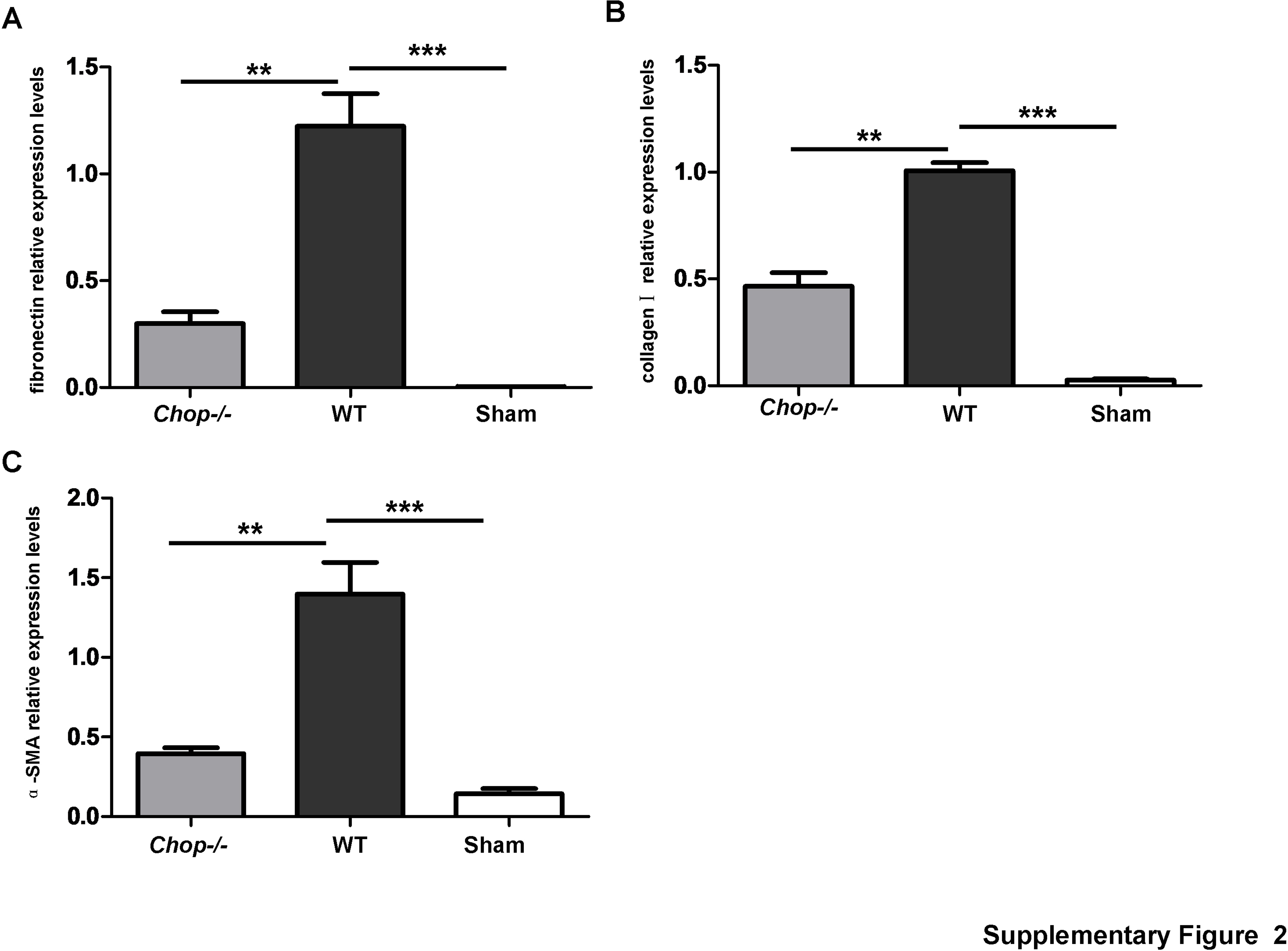

Supplement: Supplementary Figure 2 [file cddis2015206x2.tif]

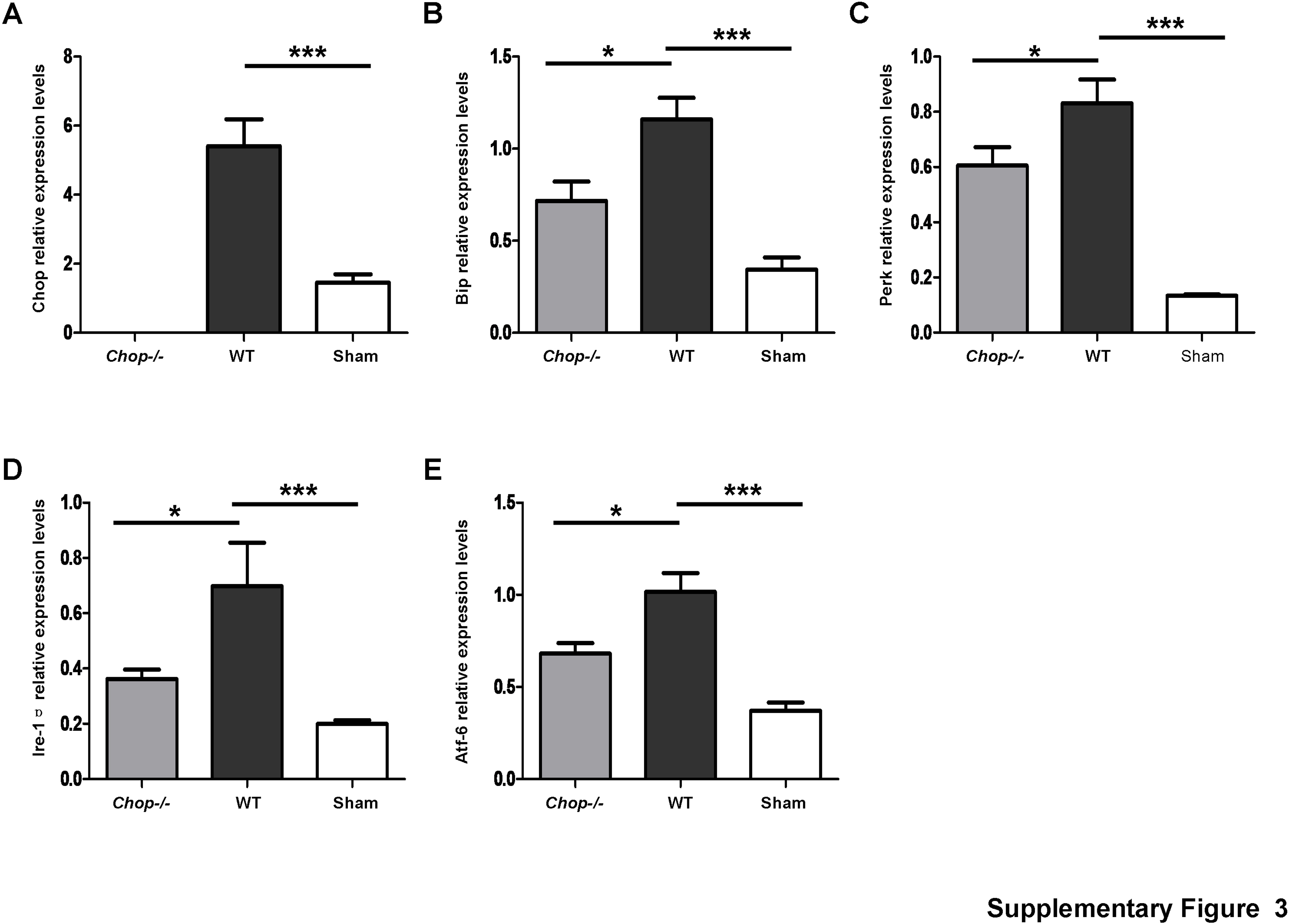

Supplement: Supplementary Figure 3 [file cddis2015206x3.tif]

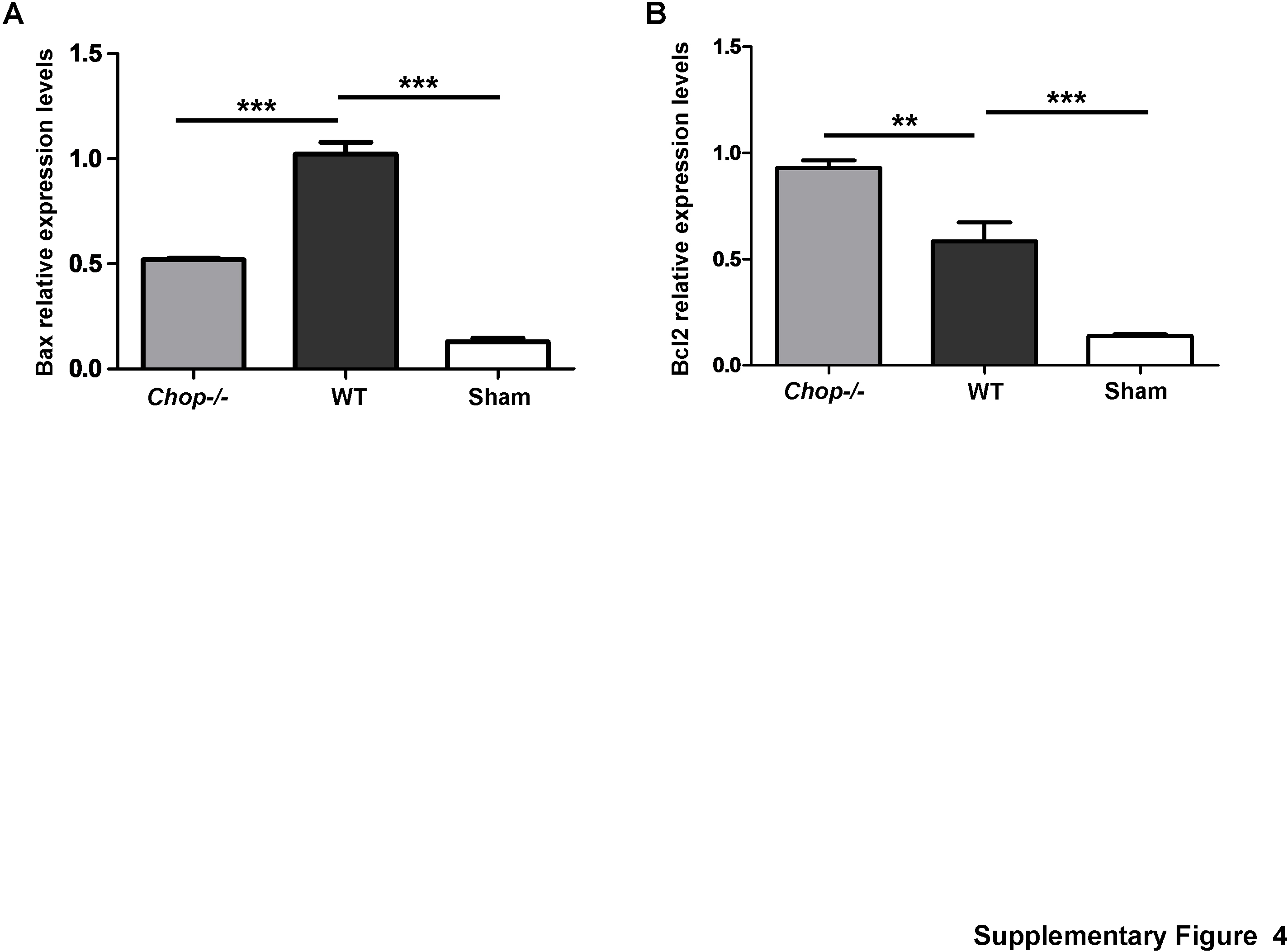

Supplement: Supplementary Figure 4 [file cddis2015206x4.tif]

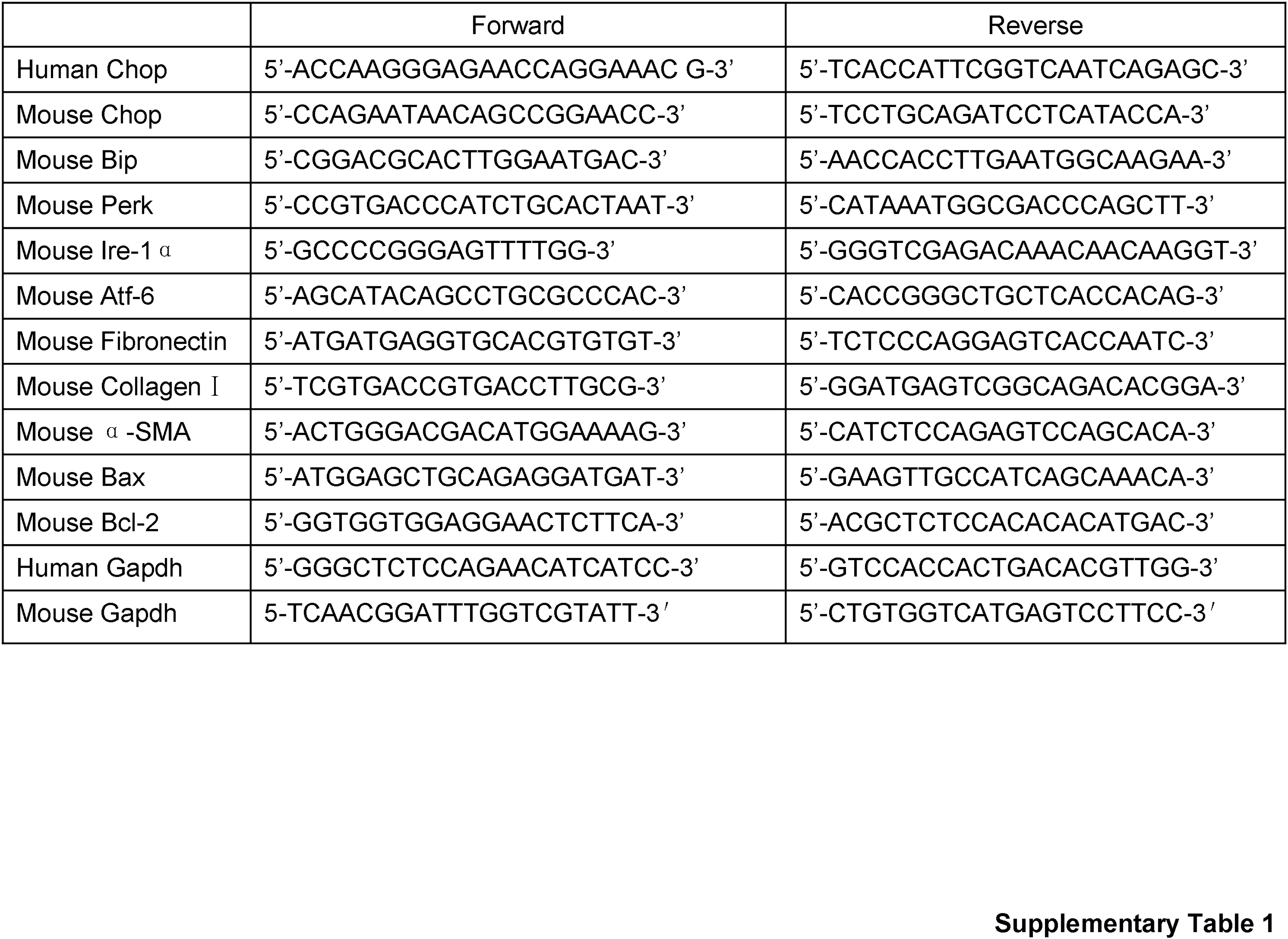

Supplement: Supplementary Table 1 [file cddis2015206x5.tif]
