# Supplementary material for: Chop deficiency prevents UUO-induced renal fibrosis by attenuating fibrotic signals originated from Hmgb1/TLR4/NFκB/IL-1β signaling
Source: Cell Death Dis. 2015 Aug 6;6(8):e1847–. doi: 10.1038/cddis.2015.206 (PMC4558499; doi:10.1038/cddis.2015.206)
Supplement: Supplementary Table and Figure Legends [file cddis2015206x6.doc]

**Supplementary Table 1: Primers for RT-PCR analysis.**

**Supplementary Figure 1: UUO-induced renal fibrosis was associated with the upregulation of mRNA levels for ER stress markers.**

mRNA levels following 14 days of UUO induction for Chop (A), Bip (B), Perk (C), Ire-1α (D), and Atf-6 (E).

**Supplementary Figure 2: Deficiency of *Chop* attenuates mRNA levels for fibrotic markers.**

Comparison of mRNA levels for Fibronectin (A), CollagenⅠ(B) andα-SMA (C) between WT and Chop^-/-^ mice following 14 days of UUO indcution.

**Supplementary Figure 3: Comparison of mRNA levels between UUO-induced WT and Chop deficient mice for Chop (A), Bip (B), Perk (C), Ire-1α (D) and Atf-6 (E).**

**Supplementary Figure 4: Loss of Chop represses UUO-induced Bax mRNA (A), but enhances anti-apoptosis factor, Bcl-2 mRNA.**
